# Genome-wide identification and tissue expression pattern analysis of *TPS* gene family in soybean (*Glycine max*)

**DOI:** 10.3389/fpls.2024.1487092

**Published:** 2024-09-26

**Authors:** Huanli Li, Xiaoling Zhang, Qinli Yang, Xiaoxia Shangguan, Yanbin Ma

**Affiliations:** Cotton Research Institute of Shanxi Agricultural University, Yuncheng, China

**Keywords:** *Glycine max*, TPS gene family, phylogenetic analysis, duplicated events, RNA-seq

## Abstract

The terpene synthase (TPS) plays a pivotal roles in plant growth, development, and enhancing resilience against environmental stresses. Despite this, the bioinformatics analysis of the *TPS* family gene in soybean (*Glycine max*) is lacking. In this study, we investigated 36 GmTPS members in soybean, exhibiting a diverse range of protein lengths, spanning from 144 to 835 amino acids. A phylogenetic tree was constructed from these *GmTPS* genes revealed a classification into five distinct subgroups: Group1, Group2, Group3, Group4 and Group5. Notably, within each subgroup, we identified the motifs of GmTPS proteins were similar, although variations existed among different subfamilies. Gene duplication events analysis demonstrated that *TPS* genes expand differently in *G. max*, *A. thaliana* and *O. sativa*. Among, both tandem duplication and Whole genome duplication contributive to the expansion of *TPS* genes in *G. max*, and Whole genome duplication played a major role. Moreover, the cis-element analysis suggested that *TPS* is related to hormone signals, plant growth and development and environmental stress. Yeast two-hybrid (Y2H) assay results indicated TPS protein may form heterodimer to function, or may form complex with P450 proteins to function. RNA-seq results revealed a higher expression of most *GmTPS* genes in flowers, suggesting their potential contribution to flower development. Collectively, these findings offer a provide a holistic knowledge of the TPS gene family in soybean and will facilitate further characterization of *TPSs* effectively.

## 1 Introduction

Terpenoids, also referred to as isoprenoids, are abundant natural products, and more than 80,000 terpenoids and their derivatives have been found so far, widely existing in plants, fungi, bacteria and insects (Realdon, 1960). TPS proteins are widely found in *algae*, bryophytes, ferns, monocotyledons and dicotyledons. Kaul et al. (2000) cloned the first TPS enzymes coding gene *AtTPS1* in *A. thaliana*, with the development of modern sequencing technologies, and more and more *TPS* genes were identified in plant genomes. For instance, there are 33 *TPS* genes in *Arabidopsis* (Aubourg et al., 2002; Yang et al., 2012), 53 in rice (Yang et al., 2012), 12 in *populus* (Yang et al., 2012), 8 in *potato* (Xu et al., 2017), 9 in *B. distachyon* (Wang et al., 2019), 34 in *d. officinale* (Yu et al., 2020), 80 in *camellia* (Zhou et al., 2020), 26 in *aloes* (Li et al., 2021), 58 in *l. chinense* (Cao et al., 2023) and 16 in *A. hypogaea* (Zhong et al., 2024). Notably, many TPS genes are also found in bacteria (Jia et al., 2019).

Previous research has categorized TPS proteins into 7 subfamilies: TPS-a–TPS-h. Specifically, TPS-a, TPS-b and TPS-g are present in angiosperms; TPS-c, closely related to TPS-e/f, is related in diterpenoid synthase production and is found in gymnosperms. Meanwhile, TPS-e/f is present in vascular plants (Newman and Chappell, 1999; Chen et al., 2011).

The regulation of *TPS* gene expression is influenced by various hormonal and environmental stresses. Such as, MeJA treatments upregulate most *CsTPS* genes expression (Zhou et al., 2020), while osmotic stress and heat stress induce *TPS* genes upregulation in roses (Yan et al., 2022). Transgenic studies show that *TPS* overexpression can enhance stress tolerance in crops like rice and *Arabidopsis*. For instance, *OsTPS1* overexpression improves rice low temperature tolerance (Ge et al., 2008) and *AhTPS9* overexpression enhances *Arabidopsis* cold tolerance (Zhong et al., 2024). Similarly, *ScTPS1* overexpression in potatoes improves drought tolerance (Yeo et al., 2000). Moreover, *OsTPS46* confers natural resistance to bird cherry-oat aphid (Sun et al., 2017), while *OsTPS24* showed no significant inhibitory activity against Magnaporthe oryzae (Yoshitomi et al., 2016). In soybeans, *GmAFS* have defensive effects against nematodes and insects (Lin et al., 2017).

Despite the importance of *TPS* genes in stress resistance, their functions in soybean remain largely unexplored. Here, we carried out a bioinformatics analysis of the *TPS* gene family in soybean, examining phylogenetic relationships, gene structures, duplication events, gene collinearity, and protein interaction networks. The tissue expression of *GmTPS* in the 6 tissues of root, young leaf, pod shell, flower, seed and nodule unravel their key regulational roles during soybean development. This study offers valuable insights and theoretical support for understanding the roles of *GmTPS* genes in soybean stress resistance.

## 2 Materials and methods

### 2.1 Data sources and identification of TPSs in soybean

The genome data were downloaded from the Soybean database (https://www.soybase.org/dlpages/). The AtTPSs and OsTPSs protein sequences were downloaded from TAIR and RGAP, respectively. The hidden Markov Model (HMM) file of PF01397, PF03936 and PF19086 were downloaded from InterPro database (Paysan et al., 2023). Utilizing HMMER 3.0, we screened for TPS proteins within soybean (E-value <= 1e-5, similarity > 50%) (Mistry et al., 2013). Additionally, we employed the BLASTP method (Camacho et al., 2009) to search GmTPS protein sequences using AtTPS and OsTPS proteins as references (E-value <= 1e-5, similarity > 50%). Subsequently, the identified candidate TPS protein sequences underwent domain verification, adopting the analysis approach outlined by Xu et al. (2017). We filtered the longest transcript using the R package seqfinder (https://github.com/yueliu1115/seqfinder).

### 2.2 Evolutionary trees are constructed of GmTPS, AtTPS and OsTPS

We employed the Muscle software (Edgar, 2004) for multiple sequence alignment of GmTPS, AtTPS and OsTPS proteins, and constructed a phylogenetic tree using IQ-TREE (Nguyen et al., 2015). The tree was visualized using the R package ggtree (Yu et al., 2017).

### 2.3 The cis-elements analysis of GmTPS genes

The 2 kb promoter region sequences upstream of the *GmTPS* gene were extracted using a Python program and submitted to the PlantCare (https://bioinformatics.psb.ugent.be/webtools/plantcare/html/) database for cis-element prediction (Lescot et al., 2002). All results were visualized in R software.

### 2.4 Analysis of chromosome distribution, gene duplication events, and selection pressure

The chromosomal distribution of *GmTPS* genes was derived from the soybean genome annotation information. MCScanX software (Wang et al., 2012) was utilized for gene duplication and colinearity analysis, identifying duplication types such as tandem (TD), and whole genome (WGD). For interspecies collinearity analysis and visualization, JCVI software (Tang et al., 2008) was employed. JCVI software was used for interspecies collinearity analysis and visualization (Tang et al., 2008). ClustalW software was used to align the protein sequences and CDS sequences of *TPS* genes with gene duplication (Thompson et al., 2003). KaKs_Calculator software was used to calculate the synonymous substitution rate (synonymous, Ks), nonsynonymous substitution rate (nonsynonymous, Ka) and evolutionary ratio (Ka/Ks) between *TPS* genes duplicate gene pairs (Zhang, 2022).

### 2.5 TPS protein interaction network analysis

The GmTPS proteins interaction network were predicted based on the AraNet2 database (Lee et al., 2015).

### 2.6 Tissue expression pattern analysis of GmTPS genes using RNA-seq

The transcriptome data of soybean under different tissues and development stages from the Soybean database (https://www.soybase.org/dlpages/). The expression data were visualized using the R package Pheatmap (Kolde and Kolde, 2015).

### 2.7 Yeast two-hybrid assays

The CDS sequence of the *Glyma.07G192800* and *Glyma.15G263300* were cloned into the pGBKT7 vector (BD-TPS); the CDS sequence of the Glyma.12G140600, Glyma.09G029400, Glyma.20G074400 and Glyma.01G153300 were cloned into the pGADT7 vector (AD-TPSs or AD-p450s). Yeast transformants with empty pGBKT7 and AD-TPSs or AD-P450s; yeast transformants with empty pGADT7 and BD-TPSs were used as the negative control. The positive control: AD-T + BD-53. Yeast transformants with AD-TPSs, BD-TPSs and AD-p450s were used to identify the TPSs interact with other TPS proteins or P450 proteins.

## 3 Results

### 3.1 Identification of TPS members in soybean

Here, a total of 36 TPS members in soybean were identified by HMMER and BLASTP methods (**Table 1**). The length of the GmTPSs protein sequence ranged from 144 (scaffold_311) to 835 (Glyma.08G163900.1); the molecular weight ranged from 16.3 (scaffold_311) to 95.6 KDa (Glyma.13G183600.1); the PI ranged from 4.27 (Glyma.13G304700.1) to 8.45 (Glyma.20G248300.1) (**Table 1**). It’s worth noting that 86% of GmTPS have isoelectric points less than 7 (**Table 1**), it is suggest that most *GmTPS* genes may are acidic proteins.

**Table 1:** Physical and chemical property analysis of TPS family genes in soybean (*Glycine max*).

Gene ID	Chr	Start	End	Amino acid length	MW	pI	Hydrophobicity
Glyma.03G154400.1	Gm03	36951469	36958622	769	87865.15	5.68	−0.28
Glyma.03G154700.1	Gm03	36991751	37001866	816	93579.29	6.70	−0.36
Glyma.06G291800.1	Gm06	48044193	48047197	324	37696.52	4.81	−0.24
Glyma.06G302200.1	Gm06	49127197	49132358	598	68742.52	6.68	−0.31
Glyma.07G187600.1	Gm07	35502526	35506056	574	65884.17	5.58	−0.26
Glyma.07G187700.1	Gm07	35531688	35536600	589	67601.58	6.05	−0.24
Glyma.07G192800.1	Gm07	36063173	36066134	380	44365.98	8.02	−0.39
Glyma.08G061600.1	Gm08	4751072	4754299	292	33490.34	5.43	−0.18
Glyma.08G163900.1	Gm08	12901875	12908991	835	95150.30	6.60	−0.18
Glyma.09G122500.1	Gm09	29418942	29427117	603	69487.15	6.36	−0.41
Glyma.10G297200.1	Gm10	51407332	51408418	225	25981.68	6.42	−0.32
Glyma.12G101700.1	Gm12	8981010	8985722	377	43496.38	5.65	−0.03
Glyma.12G102000.1	Gm12	9043334	9048807	603	69766.91	7.08	−0.32
Glyma.12G138100.1	Gm12	16508959	16517078	531	61954.20	5.62	−0.27
Glyma.12G138600.1	Gm12	16685178	16690007	554	64037.47	5.91	−0.19
Glyma.12G138800.1	Gm12	16733572	16741825	501	58069.48	6.38	−0.34
Glyma.12G140600.1	Gm12	17392329	17399822	561	65363.99	5.37	−0.24
Glyma.12G179500.1	Gm12	33977724	33980896	421	48739.09	6.65	−0.30
Glyma.12G197400.1	Gm12	35854107	35858237	569	65013.70	6.91	−0.26
Glyma.12G197500.1	Gm12	35869161	35874097	585	67079.78	5.91	−0.27
Glyma.12G216200.1	Gm12	37539186	37543933	565	65213.59	5.64	−0.27
Glyma.13G183600.1	Gm13	29714220	29722406	832	95610.00	7.08	−0.27
Glyma.13G250400.1	Gm13	35790066	35794185	535	62338.13	5.87	−0.29
Glyma.13G285100.1	Gm13	38608137	38609427	256	29739.02	6.18	−0.31
Glyma.13G285200.1	Gm13	38611052	38614182	566	64535.43	5.56	−0.24
Glyma.13G304500.1	Gm13	40139730	40145370	580	66250.99	6.46	−0.26
Glyma.13G304600.1	Gm13	40155002	40157401	310	36171.91	6.19	−0.52
Glyma.13G304700.1	Gm13	40159803	40161491	233	27231.76	4.27	−0.33
Glyma.13G304800.1	Gm13	40161767	40162729	176	20013.88	6.51	−0.36
Glyma.13G321100.1	Gm13	41540416	41543961	569	65844.55	6.51	−0.26
Glyma.15G263300.1	Gm15	49639950	49645729	424	48964.87	5.06	−0.25
Glyma.19G156800.1	Gm19	41726563	41730894	291	33626.12	7.13	−0.35
Glyma.19G157000.2	Gm19	41763615	41774896	817	93957.76	6.64	−0.36
Glyma.20G074400.1	Gm20	26716996	26720775	607	70199.87	5.94	−0.46
Glyma.20G248300.1	Gm20	47752687	47755022	297	34026.17	8.45	−0.32
Glyma.U032900.1	scaffold_311	1109	2723	144	16302.60	6.09	−0.27

### 3.2 Phylogenetic analysis of TPSs

**Figure 1:**
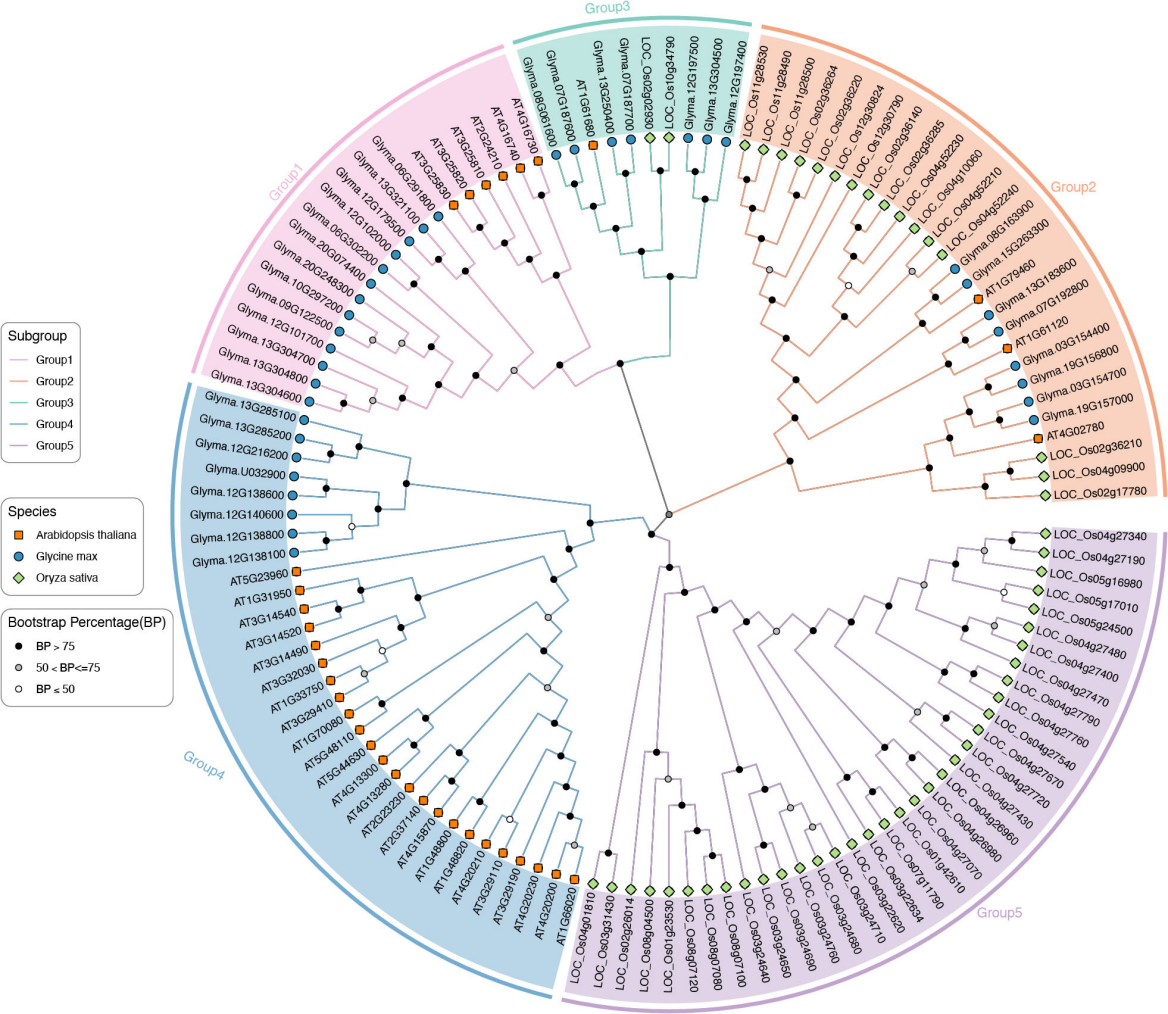
Phylogenetic tree of 36 GmTPSs, 33 AtTPSs and 53 ScTPSs. The evolutionary tree was constructed by the maximum likelihood method. Pink, orange, sky blue, blue and purple represent the subfamily of Group1, Group2, Group3, Group4 and Group5, respectively.

To deeper understanding the evolutionary dynamics of GmTPSs, we constructed a phylogenetic tree that comprehensively encompasses 36 GmTPSs, along with 33 AtTPSs and 53 OsTPSs. The *TPS*s members could be grouped into Group1, Group2, Group3, Group4 and Group5 (**Figure 1**). Group5 contained the largest number of 35 *TPS*s, while Group3 contained the smallest number of 10 *TPS*s. Group1, Group*2*, and Group4 contained the number of 19, 27 and 32 *TPS*s, respectively. Interestingly, no *GmTPS*s and At*TPS*s member was found in the subgroup of Group5, and Group1 and Group4 contain only GmTPS and AtTPS members (**Figure 1**). These observations provide insights into the evolution of the *TPS* gene family.

### 3.3 Gene structure and motifs analysis of GmTPS

**Figure 2:**
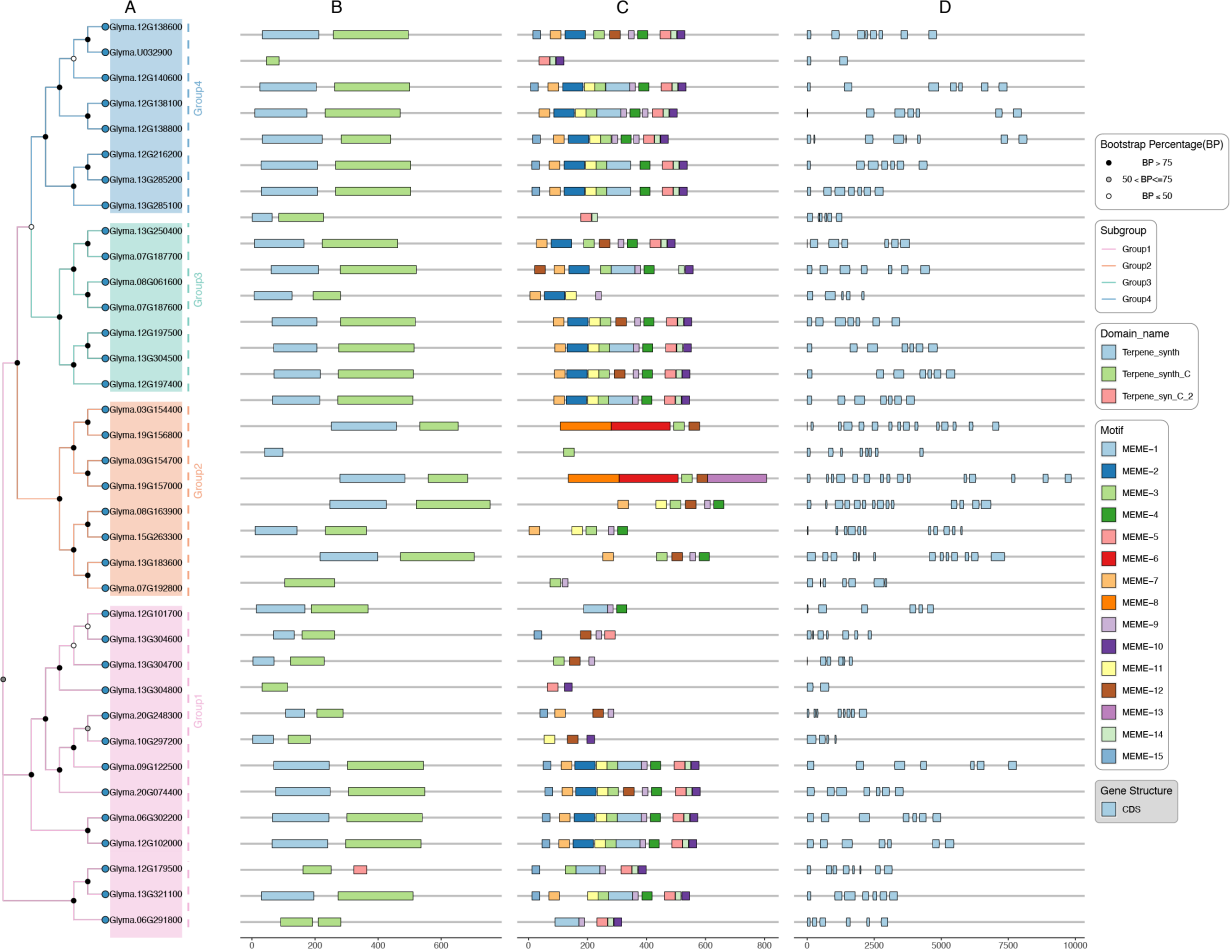
The domains, motifs and gene structure of GmTPSs were analyzed. **(A)** Phylogenetic tree, **(B)** The domains were predicted by NCBI-CDD. **(C)** The motifs of GmTPSs were predicted by MEME. **(D)** Gene structure of *GmTPS*.

To comprehend the diversity of *GmTPS* genes, we analyzed the gene structures, and conserved domain and conserved motif in GmTPS, the results are presented in **Figure 2**. Most of the GmTPS members contained two conserved domains, the Terpene_synth domain at the N-terminal and the Terpene_synth_C domain at the C-terminal (**Figure 2**). It is worth noting that Glyma.U032900 and Glyma.07G192800 consist only of the Terpene_synth_C domain; Glyma.19G156800 consist only of the Terpene_synth domain. Interestingly, Glyma.06G291800 contain two Terpene_synth_C domains (**Figure 2**). Except for the Terpene_synth_C domains, Glyma.12G179500 also contains Terpene_syn_C_2 domain.

On the other hand, in the same subgroups, we found the conserved motifs of GmTPS were similar, although variations existed among different subfamilies (**Figure 2**). For example, Group4 Subgroup members Glyma.12G138600, Glyma.12G140600, Glyma.12G138800, Glyma.12G216200 and Glyma.13G285200 contain conserved motif 1, motif 2, motif 3, motif 5, motif 6, motif 7, motif 10, motif 11, motif 14 and motif 15; Glyma.12G197500 and Glyma.12G197400 contain conserved motif 1, motif 2, motif 3, motif 4, motif 6, motif 7, motif 9, motif 10 motif 11 and motif 14 (**Figure 2**). These diverse motifs reflect the functional diversity of GmTPS proteins. On the other hand, we found that most *TBS* genes contain multiple introns, except for *Glyma.U032900* and *Glyma.13G304800*, which contain only one intron (**Figure 2**). The variations of motifs and gene structure may contribute to the diverse biological functions of *GmTPSs*.

### 3.4 Duplication events analysis of GmTPS genes

**Figure 3:**
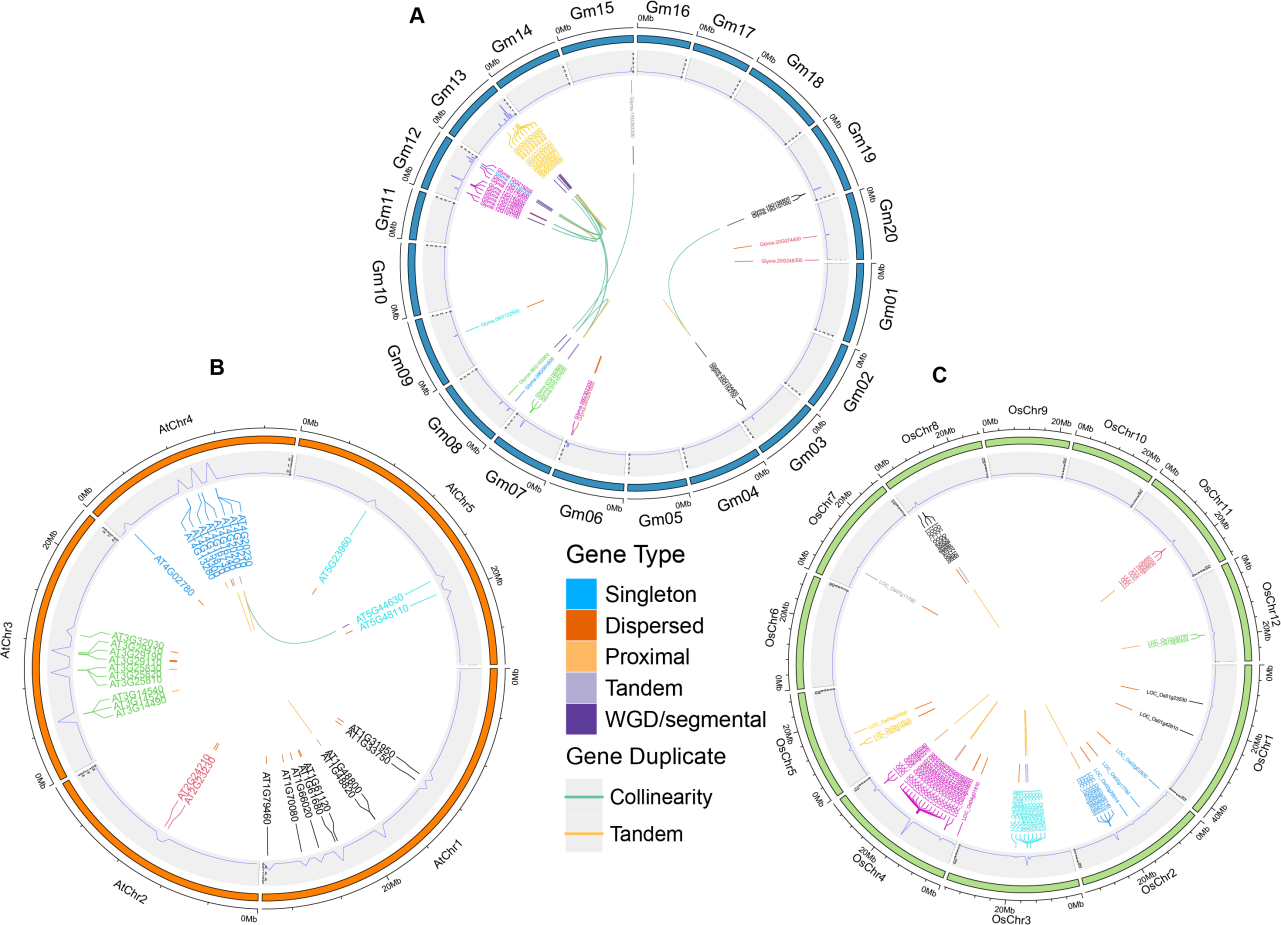
The chromosome location and duplicated gene pair of *TPS* genes in three species including **(A)** soybean, **(B)** *Arabidopsis* and **(C)** rice. The duplicate gene types were displayed in different color. WGD and TD events are shown in orange and blue, respectively.

According to GFF files, we analyzed the gene distribution of 36 *GmTPS*. The 36 *GmTPS* genes were distributed on 10 chromosomes, while no *GmTPS* genes were distributed on chromosomes Chr1, Chr2, Chr4, Chr5, Chr10, Chr11, Chr14, Chr16, Chr17 and Chr18 (**Figure 3**). Notably, the most *GmTPS* genes were distributed at chr12 and chr13, with 10 and 19 respectively, forming gene clusters. Both Chr9 and Chr15 contain one *GmTPS* members, while both Chr3, Chr6, Chr8, Chr19 and Chr20 contain two *GmTPS* members (**Figure 3**).

TD and WGD drive the expansion of the gene family (Freeling, 2009; Panchy et al., 2016). Therefore, we explored the duplication events of *TPS* genes in soybean, *Arabidopsis* and rice. In this study, 16 WGD gene pairs and 18 TD gene pairs were confirmed in soybean, *Arabidopsis* and rice (**Figures 3A–C**; **Supplementary Table 1**). Overall, in soybean, *Arabidopsis* and rice, 10 (27.78%), 18 (60%) and 16 (30.19%) *TPS* genes were confirmed to be TD, and 23 (63.89%), 2 (6%) and 0 (0%) *TPS* genes were found to be WGD, respectively (**Figures 3A–C**; **Supplementary Table 1**). These data show that both TD and WGD contributive to the expansion of *TPS* genes in soybean, and WGD played a major role. However, in *Arabidopsis*, TD and WGD both promoted the expansion of *TPS* genes, and TD plays a leading role. Interestingly, in rice, only TD replication events were found. These data suggest that *TPS* genes expand differently in soybean, *Arabidopsis* and rice.

### 3.5 Collinearity analysis of *GmTPS* genes

**Figure 4:**
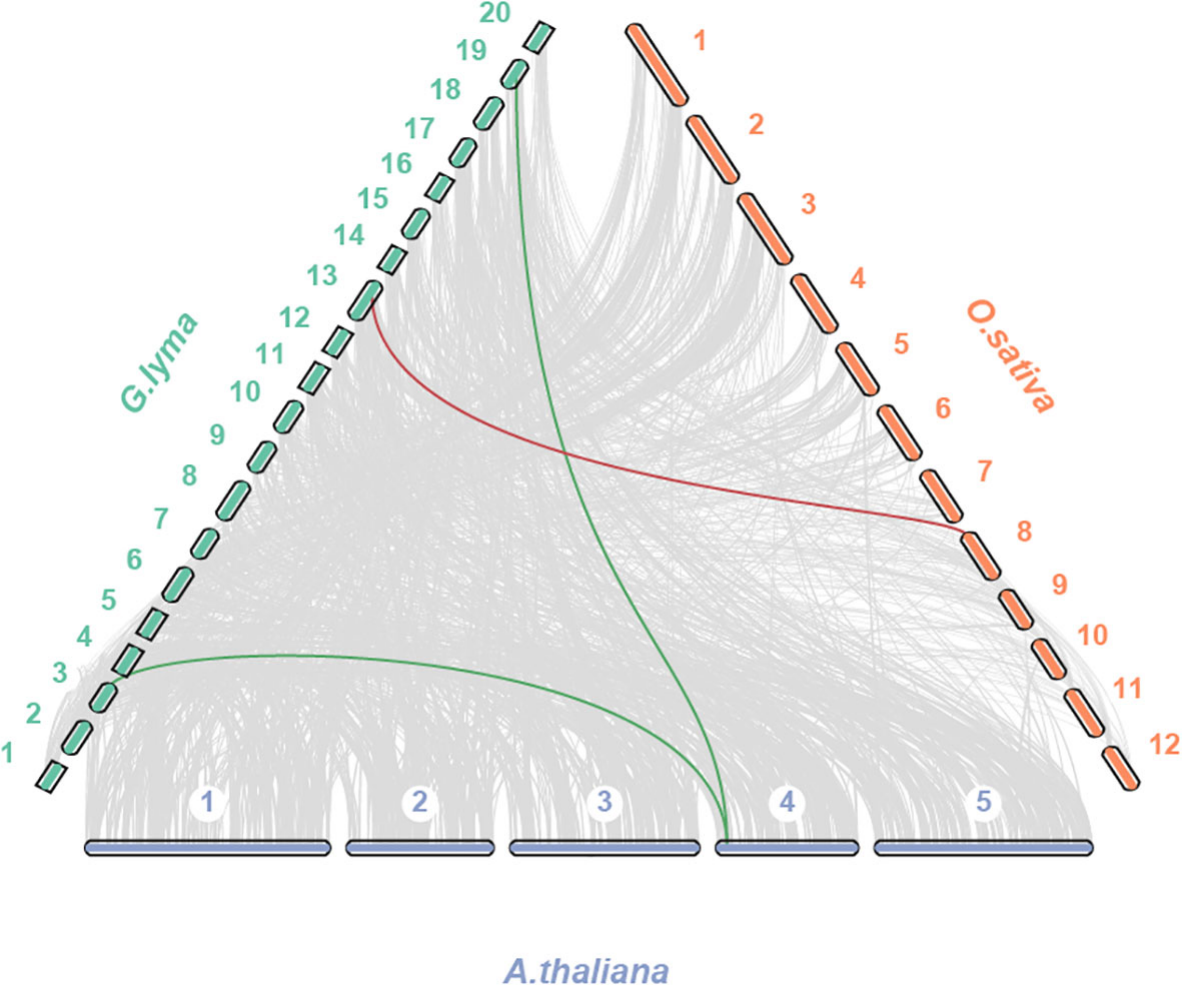
Syntenic analysis of *GmTPS* genes between and *Arabidopsis* and rice. The collinear blocks and *TPS* homologous genes pairs were shown by gray and red lines, respectively.

To deeper investigate the homology of the *TPS* gene family in *G. max*, we conducted a comparative analysis of *TPS* gene collinearity between *G. max* and two model organisms, *Arabidopsis* and rice. Our findings revealed that 2 *AtTPS* and 1 *OsTPS* were homologous gene pairs with *GmTPS* (**Figure 4**). To gain insights into the evolutionary pressures of *TBS* genes in soybean, *Arabidopsis* and rice, we employed DnaSP software to calculate Ka/Ks ratios. Here, we found that the Ka/Ks value of all *TPS* duplication gene pairs is less than 1 in soybean, *Arabidopsis* and rice (**Supplementary Table 1**). These results suggest that *GmTPS*, *AtTPS* and *OsTPS* genes were under purifying selection.

### 3.6 Cis-elements analysis of GmTPS

**Figure 5:**
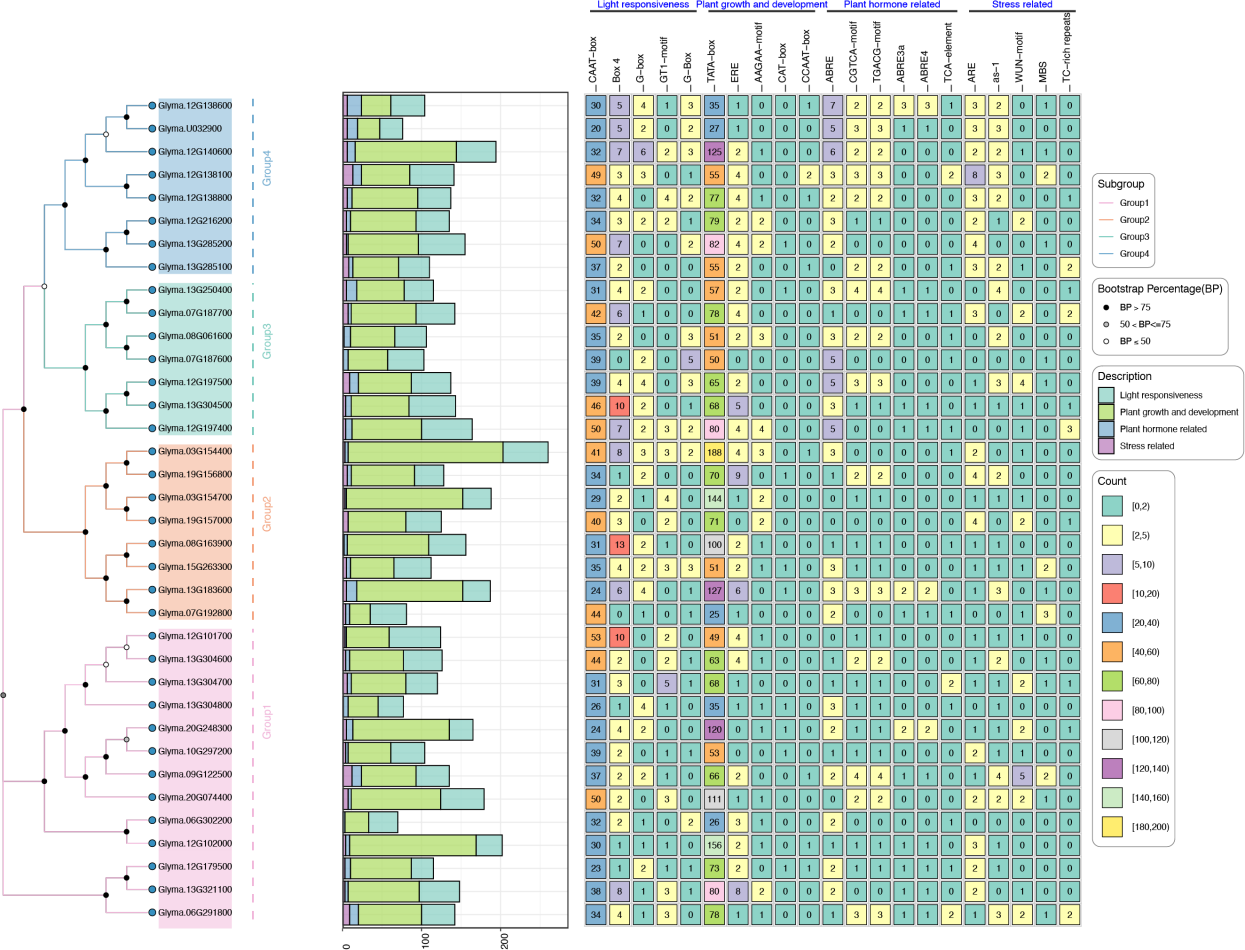
Cis-elements analysis of *TPS* genes in *G. max*.

To unravel the may regulatory mechanisms of *GmTPS genes*, we analyzed their 2k promoter regions, uncovering a diverse array of 21 cis-elements (**Figure 5**). These elements encompass various functional categories, including light response-related cis-elements (CAAT-box, Box-4, e.g); plant growth and developmental; phytohormone (ABRE, CGTCA-motif, TGACG-motif and TCA-element) and stress response related cis-elements (ARE, as-1, WUN-motif, MBS and TC-rich repeats) (**Figure 5**). Interestingly, Group4 *GmTPS* genes contain ABRE, CGTCA-motif and TGACG-motif cis-elements (**Figure 5**), hinting that *GmTPS* genes may be involved in ABA and JA signaling pathways. In addition, *Glyma.13G285100* contains CGTCA-motif, TGACG-motif and TCA-element, suggesting that it may antagonistically participate in SA and JA signaling pathways (**Figure 5**). It’s worth noting that *Glyma.19G157000* contains a large number of light, plant growth and developmental and stress response cis-elements, while no hormone response cis-elements are found. Furthermore, our analysis revealed that *Glyma.07G192800*, *Glyma.12G138100*, *Glyma.15G263300*, and *Glyma.09G122500* contain varying numbers of MBS cis-elements, indicative of potential roles in drought signaling pathways (**Figure 5**). The above data indicates that *GmTPS* may have complex regulatory functions.

### 3.7 Interaction network of GmTPS proteins

**Figure 6:**
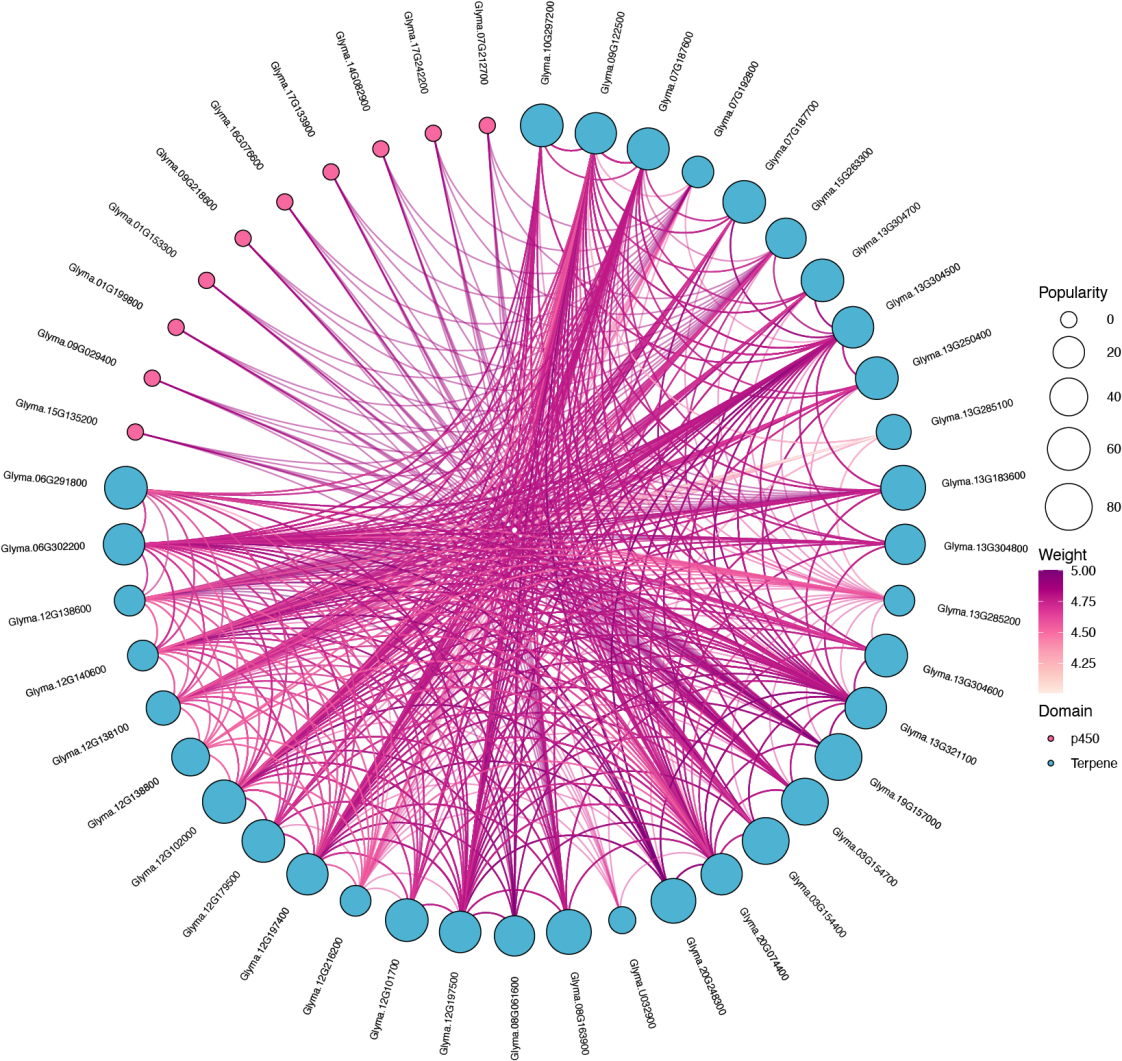
The protein-protein interaction network of TPSs protein in *G. max*.

To gain deeper insights into the functional roles and regulatory intricacies within the *GmTPS* gene family, we leveraged the AraNet2 database to analyze and predict a protein-protein interaction network. This analysis revealed extensive interconnectivity among the GmTPS proteins, with nearly all members engaging in interactions (**Figure 6**). Interestingly, we found that some GmTPS proteins can interact with P450 proteins, suggesting potential functional crosstalk or coordinated activities between these two protein classes (**Figure 6**).

**Figure 7:**
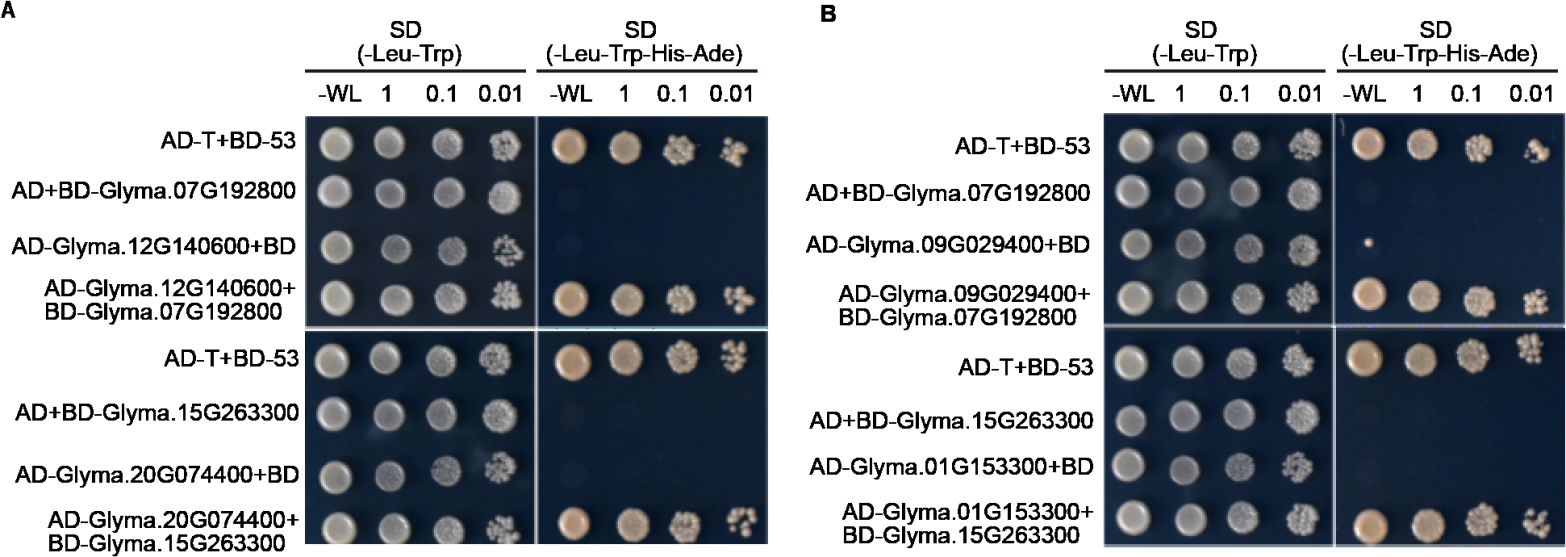
Y2H assays show TPS proteins can interact with other TPS proteins or P450 proteins. **(A)** The image showed the results of TPS proteins can interact with other TPS proteins. **(B)** The image showed the results of TPS proteins can interact with P450. The positive control: AD-T + BD-53.

In addition, we selected Glyma.07G192800 and Glyma.15G263300 proteins to verify whether TPS proteins can interact with other TPS proteins or P450 proteins. To this end, we used the Y2H assay. As data shown in **Figure 7**, we found that Glyma.07G192800 can interact with Glyma.12G140600, and Glyma.15G263300 can interact with Glyma.20G074400 (**Figure 7**). Moreover, we found that Glyma.07G192800 can interact with P450 proteins Glyma.09G029400, and Glyma.15G263300 can interact with P450 proteins Glyma.01G153300 (**Figure 7**). These data indicate that TPS protein may form heterodimer to function, or may form complex with P450 protein to function.

### 3.8 The tissues expression analysis of GmTPS

**Figure 8:**
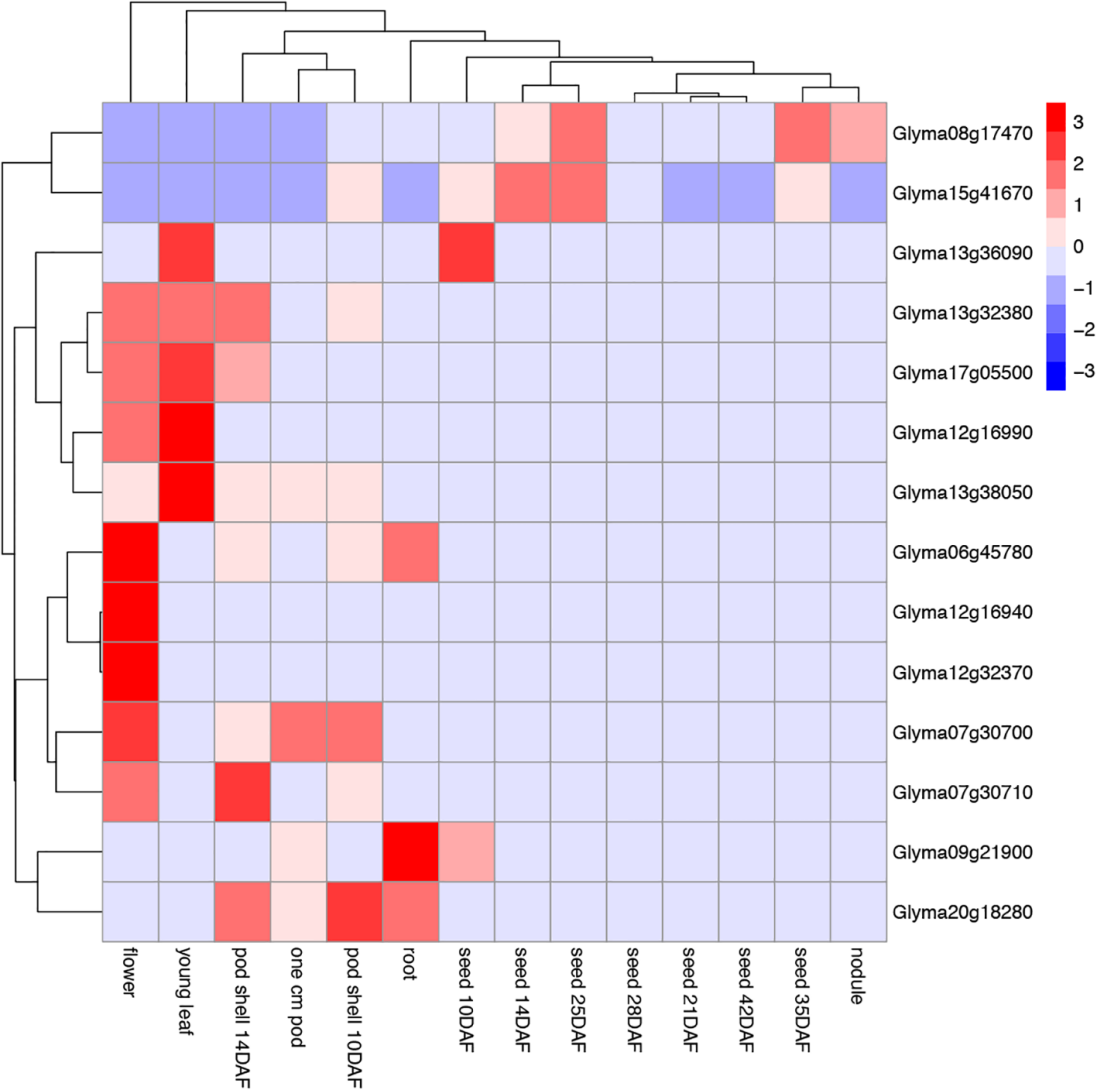
The heat map of the tissue expression of *GmTPS* genes in different tissues and development stages (flower, young leaf, pod shell 14DAF, one cm pod, pod shell 10DAF, root, seed 10DAF, seed 14DAF, seed 25DAF, seed 28DAF, seed 21DAF, seed 42DAF, seed 35DAF and nodule). The data are shown in a heatmap with gene expression in different tissues and development stages with row-scaled FPKM values.

To further explore the functions of *GmTPS*, we analyzed its expression pattern based on RNA-seq data. *Glyma.06g45780*, *Glyma.12g16940*, *Glyma.12g32370* and *Glyma.07g30700*, expressed higher in flower than other tissues. We speculate that these GmTPS genes may synergistically regulate soybean growth and development (**Figure 8**). And Glyma.12g16940 and *Glyma.12g32370* are expressed only in flower. Additionally, these GmTPSs no expression of *Glyma.13g32380*, *Glyma.17g05500*, *Glyma.12g16990*, *Glyma.13g38050*, *Glyma.06g45780*, *Glyma.12g16940*, *Glyma.12g32370*, *Glyma.07g30700*, *Glyma.07g30710* and *Glyma.20g18280* are detected in the organs of seed and nodule. On the other hand, *Glyma.08g17470* and *Glyma.15g41670* are widely expressed in root, young leaf, pod shell, flower, seed and nodule (**Figure 8**). The result indicate that *GmTPS* genes may involve in diverse aspects of plant growth and developmental processes.

## 4 Discussion

Terpenoids, widely existing in plants, fungi, bacteria and insects, and play an pivotal role in enhancing plant resistance. It is worth noting that the TPS proteins are involved in many biological processes, such as low-temperature stress adaptation, drought stress adaptation, salt stress adaptation, and responses to phytohormonal and insect resistance (Yeo et al., 2000; Li et al., 2011; Huang et al., 2018; Ge et al., 2008; Zhou et al., 2020; Yan et al., 2022; Zhong et al., 2024). While the *TPS* gene family has been found in many species, but, the whole-genome identification and bioinformatics analysis of *TPS* gene family in soybean is lacking. In this study, we systematically analyzed the *TPS* gene family in soybean using bioinformatics methods and identified a total of 36 *TPS* genes (**Table 1**). *TPS* genes, which are ubiquitous among plant species, such as 33, 53, 12, 8, 80, 26, 58 and 16 *TPS* genes were found in *Arabidopsis*, rice, *populus*, *potato*, *d. officinale*, *camellia*, *aloes*, *l. chinense* and *A. hypogaea* (Aubourg et al., 2002; Yang et al., 2012; Xu et al., 2017; Zhou et al., 2020; Li et al., 2021; Cao et al., 2023 and Zhong et al., 2024). It is not difficult to see that the member of TPS genes in different plants varies greatly. These results also showed that TPS genes may not only be functionally conserved, but also functionally differentiated in different plants.

Prior research has categorized TPS proteins into seven distinct subfamilies: TPS-a through TPS-h. are predominantly found in angiosperms, while TPS-c is specific to gymnosperms, and TPS-e/f occurs in vascular plants (Newman and Chappell, 1999; Chen et al., 2011). Phylogenetic tree showed that the 122 TPS proteins in GmTPSs, AtTPSs and OsTPSs can be divided into five groups (**Figure 1**). Interestingly, Group5 only exists in the rice, and Group1 and Group4 only exists in soybean and *Arabidopsis*. These observations may provide insights into the evolution and diversification of the *TPS* gene family in monocotyledons and dicotyledons.

During the progress of evolution, TD and WGD events played a key role in the expansion of gene families, new genes and novel functions (Freeling, 2009; Panchy et al., 2016). In our study, we observed rapid expansion of the Group4 subgroup in *G. max* due to recent TD and WGD, while the Group5 subgroup experienced rapid expansion in *O. sativa* for the TD. Overall, the number of WGD genes was the largest, indicating that WGD was found to be the predominant mechanism driving the evolution and expansion of the *TPS* gene family in *G. max*. This investigation offers profound insights into the evolutionary journey and expansion patterns of the *TPS* gene family across diverse plant species.

Numerous studies found the important role of *TPS* genes in mediating plant responses to different hormone signals, and abiotic and biotic stress (Yeo et al., 2000; Li et al., 2011; Huang et al., 2018; Ge et al., 2008; Zhou et al., 2020; Yan et al., 2022; Zhong et al., 2024). For instance, *OsTPS1* overexpression in rice boosts trehalose levels, enhancing resilience against low temperatures (Ge et al., 2008). Similarly, *TaTPS11* overexpression in *Arabidopsis* enhances cold tolerance (Liu et al., 2019), while *ScTPS1* overexpression in tomato elevates drought tolerance (Cortina and Culiáñez-Macià, 2005). Our cis-acting element analysis revealed that *Glyma.07G192800*, *Glyma.12G138100*, *Glyma.15G263300* and *Glyma.09G122500* contain three, two, two and two MBS cis-elements, respectively (**Figure 5**). This result implies that these *GmTPS* genes may be involved in drought signaling pathways, and will be an interesting topic to explore in the future.

On the other hand, MeJA treatment transcriptionally upregulated the expression of most *CsTPS* genes (Zhou et al., 2020). Our analysis uncovered the prevalence of MYC2, ABRE, CGTCA-motif, TGACG-motif, and WUN-motif in the promoters of *GmTPS* genes, particularly *Glyma.12G138100*, *Glyma.15G263300*, and *Glyma.09G122500*, which harbor ABRE, CGTCA-motif, TGACG-motif, as-1, WUN-motif, and MBS cis-elements (**Figure 5**). Despite the scarcity of experimental evidence elucidating the intricate relationships between phytohormone signaling and terpenes biosynthesis, we hypothesize that intricate crosstalks among distinct phytohormone signaling pathways may delicately modulate terpenes biosynthesis through a myriad of transcription factors, and will an interesting topic to explore in the future.

Cytochrome P450s (CYPs) orchestrate an array of essential processes, encompassing growth, development, and the biosynthesis of secondary metabolites (Mizutani and Ohta, 2010 and 2012). For instance, P450 enzymes exhibit remarkable adaptability in modulating plant development through hormone synthesis (Schuler, 1996). Specifically, *CYP707A* play a key role in the catalytic synthesis of ABA (Saito et al., 2004), while *CYP94B3*, *CYP94C1* and *CYP74B* were related in JA biosynthesis (Li et al., 2008; Koo et al., 2011; Heitz et al., 2012). Our observations that some TPS proteins can interact with P450 proteins in soybean during the Y2H assays (**Figure 7**), suggest that TPS proteins likely form complexes with P450 proteins and participate in the growth, development, and the biosynthesis of secondary metabolites in soybean.

## 5 Conclusions

In this study, we identified 36 *TPS* members in soybean and systematically grouped them into five distinct subfamilies: Group1, Group2, Group3, Group4 and Group5. Subsequently, we demonstrated that both TD and WGD contributed significantly to the expansion of TPS genes in *Glycine max*, with WGD playing a pivotal role. Furthermore, our analysis revealed that all *GmTPS*, *AtTPS*, and *OsTP*S genes were subjected to purifying selection. Yeast two-hybrid (Y2H) assay results showed that TPS protein may form a heterodimer to function, or may form a complex with P450 protein to function. RNA-seq data displayed *GmTPS* genes are involved in soybean growth and development. This exhaustive study establishes a foundational understanding of the pivotal roles played by *GmTPS* genes in soybean.

## Data Availability

The datasets presented in this study can be found in online repositories. The names of the repository/repositories and accession number(s) can be found in the article/**Supplementary Material**.

## Supplementary material

Supplementary Data Sheet 1 The information of Ka, Ks and Ka/Ks values of duplicate gene pairs.

Supplementary Data Sheet 2 The information on primer sequences of genes involved in this study.
